# Optimization of pesticide spray parameters and analysis of key physiological indicators for control efficiency against jujube leaf black spot

**DOI:** 10.3389/fpls.2026.1742081

**Published:** 2026-02-05

**Authors:** Rui-Xia Zhang, Jun-Ya Hao, Ke Xiao, Ping Liu

**Affiliations:** 1 College of Horticulture, Hebei Agricultural University, Baoding, Hebei, China; 2 Hebei Jujube Industry Technology Research Institute, Hebei Agricultural University, Baoding, Hebei, China; 3 Research Center of Chinese Jujube, Hebei Agricultural University, Baoding, Hebei, China; 4 College of Information Science and Technology, Hebei Agricultural University, Baoding, Hebei, China; 5 Hebei Key Laboratory of Agricultural Big Data, Hebei Agricultural University, Baoding, Hebei, China

**Keywords:** correlation, disease severity grades, jujube leaf black spot disease, physiological indicators, spraying parameters

## Abstract

**Introduction:**

Jujube leaf black spot is a common foliar disease in jujube orchards. Precision spraying technology enhances pesticide utilization efficiency through accurate targeting and on-demand application, while simultaneously mitigating environmental risks.

**Methods:**

In this study, 1,000 jujube black spot leaves with different disease severity levels were selected, labeled, and photographed. Chemical treatments were applied at varying spraying distances and angles according to the disease severity, with a water control group established for comparison. The control efficiency of spraying parameters on jujube leaf black spot across different disease severity levels and their physiological effects on the leaves were measured.

**Results:**

The spraying parameters significantly influenced the control efficiency across different disease severity levels. For level 0 (prevention), a distance of 0.9 m and an angle of 0°; for level 1, a distance of 1.2 m and an angle of 0°; for level 2, a distance of 0.6 m and an angle of 40°; for level 3, a distance of 0.6 m and an angle of 20°; and for level 4, a distance of 0.9 m and an angle of 40°. The control efficiency showed a significant positive correlation with soluble sugar (SS) content and the activities of superoxide dismutase (SOD), peroxidase (POD) and phenylalanine ammonia-lyase (PAL). Among these, SOD and POD activities were identified as key physiological indicators for evaluating the physiological impact of chemical control on jujube leaf black spot across different severity levels.

**Discussion:**

By investigating the graded control efficiency of spraying parameters on jujube leaf black spot, this study screened the optimal spraying parameters for graded disease control and identified key physiological indicators for evaluating control efficiency, providing a theoretical basis for the green control of jujube leaf black spot.

## 1 Introduction

Jujube leaf black spot disease is primarily caused by *Alternaria alternata* (Fr.) Keissler (Zhang et al., 2021). It mainly affects the leaves of jujube trees. At the initial stage of infection, scattered black spots appear on the back of the leaves, which later expand into circular or irregular lesions. In severe cases, the leaves curl and fall prematurely, impacting jujube yield and fruit quality. The pathogen directly penetrates the leaf stomata. The field incubation period is 2~3 days before symptoms appear, and conidia are produced 3~4 days after lesion formation. Conidia are dispersed via air currents and rain splash, leading to multiple rounds of re-infection and disease spread. After the rainy season begins, a peak period of disease occurrence follows (Deng et al., 2024).

Spraying pesticides is a primary method for controlling jujube leaf black spot disease. It works by inhibiting spore germination, hindering mycelial growth, interfering with cellular metabolism, protecting plant tissues, and inducing plant resistance, thereby enhancing the plant’s disease resistance and reducing disease incidence (Jiang et al., 2023; Yan, 2023). With the increasing emphasis on pesticide safety and agricultural modernization, precision spraying has replaced traditional manual spraying methods (Hu et al., 2023; Song et al., 2025). Disease severity grading helps clarify the extent of disease damage, facilitating the determination of pesticide requirements in different areas and avoiding indiscriminate pesticide use. By adjusting spraying parameters such as distance and angle, precise pesticide application can be ensured, improving pesticide utilization efficiency and reducing waste and environmental pollution (Tang et al., 2025). Combining these approaches enables on-demand spraying, enhances control efficiency, and reduces agricultural production costs (Farooque et al., 2023). Although pesticides can suppress diseases and pests, they may also interfere with plant growth, cause cell membrane damage, and affect physiological processes such as osmotic regulation, antioxidant capacity, and stress resistance (Köseoğlu and Doğru, 2025; Rocha et al., 2025). Therefore, clarifying the impact of spraying parameters on leaf physiological indicators under different disease severity levels is a critical step in achieving precise pesticide application.

Common optimization methods for spraying parameters mainly include response surface methodology, intelligent optimization algorithms, and real-time monitoring with feedback control. Response surface methodology predicts the optimal parameter combinations through mathematical models, but it relies on extensive experimental data to construct surface relationships and has limited adaptability to complex nonlinear problems (Sun et al., 2018; Zhang et al., 2025a). Although intelligent optimization algorithms can globally search for optimal solutions, they involve high computational costs, strong parameter sensitivity, and their decision-making processes often lack consideration of pesticide toxicology and crop tolerance (Ijaz et al., 2024). Real-time monitoring and feedback control technologies can dynamically respond to environmental changes and improve application accuracy, but they come with high hardware costs and their stability is constrained by harsh field conditions (Islam and Hatou, 2024). The aforementioned methods primarily focus on computer models or mechanical operations and do not account for the actual impact of pesticides on plant physiology and the environment in practical agricultural production. This study proposes an optimization approach for graded spraying by investigating the effects of spraying parameters on the control efficiency and physiological responses of jujube leaves affected by black spot disease at different severity levels. Compared to existing methods, this approach can obtain optimal spraying parameters while effectively reducing the physiological damage caused by pesticides to plants. It offers a new method for achieving precise variable-rate spraying, advancing graded optimization of pesticide application, and promoting the development of eco-friendly plant protection technologies.

In the field of agricultural plant protection, existing research has predominantly focused on abiotic stress or single-disease conditions. Studies have extensively explored the correlation between plant physiological indicators and yield (Buragohain et al., 2024), the correlation between different treatments and plant physiological resistance, as well as correlations among plant physiological indicators (Ahmad et al., 2021; Shivran et al., 2024). Research on the correlation between control efficiency and physiological indicators after pesticide application further elucidates the inherent relationship between control effects and physiological responses, providing critical insights for environmentally friendly and precise control strategies. Zhang et al. (2022a) found that the dosage and type of pesticides significantly affect plant physiological indicators, while Baharim et al. (2024) observed significant correlations between physiological indicators and disease severity. These findings demonstrate the feasibility of achieving graded optimized spraying by investigating the impact of spraying parameters on plant physiology.

To this end, this study selected ‘Dongzao’ jujube leaves infected with black spot disease as the research subjects. Different spraying distances and angles were set for control treatments, and the control efficiency and physiological response characteristics of leaves at different disease severity levels after intervention were systematically analyzed. By measuring changes in lesion area, malondialdehyde (MDA) content, soluble sugar (SS) content, and the activities of key defense enzymes such as superoxide dismutase (SOD), peroxidase (POD), catalase (CAT), and phenylalanine ammonia-lyase (PAL), the effects of spraying parameters on lesion area and physiological indicators across different disease severity levels were analyzed. Furthermore, the correlation between control efficiency and physiological indicators after pesticide application was investigated to determine the optimal spraying distance and angle for different severity levels of jujube leaf black spot disease. This study aims to provide a theoretical basis for precise graded control and to contribute to the development of a more comprehensive model for plant physiological regulation.

## 2 Materials and methods

### 2.1 Experimental materials

The test materials were ‘Dongzao’ jujube leaves with black spot disease. In July 2024, diseased leaves in a jujube orchard at the Third Farm of Hebei Agricultural University (Baoshuo Road, Lianchi District, Baoding City, Hebei Province, China) were marked. During the peak incidence period of jujube leaf black spot disease in August 2024, the marked leaves were photographed and treated with fungicide. A 2500×dilution of 43% Fluopyram + Tebuconazole Suspension Concentrate (manufactured by Arysta LifeScience North America Corp.) was used for black spot disease control. In September 2024, diseased leaves of different severity grades were collected, placed in liquid nitrogen, brought back to the laboratory of Hebei Agricultural University, and stored in a -80°C freezer for subsequent determination of relevant indicators.

### 2.2 Experimental methods

#### 2.2.1 Grading of jujube leaf black spot disease

Based on national requirements for comprehensive plant disease and pest control, as well as the local standard “Technical code of practice for comprehensive control of jujube black spot”(DB65/T 4731-2023) issued by the Market Supervision Administration of Xinjiang Uygur Autonomous Region, the severity of jujube leaf black spot disease was classified into 5 grades (**Table 1**).

**Table 1:** Disease classification of jujube leaf black spot.

Disease grade	Diseased leaf area
Grade 0	No lesions on jujube leaves
Grade 1	Diseased area ≤ 20%
Grade 2	20%< Diseased area ≤ 25%
Grade 3	25%< Diseased area ≤ 50%
Grade 4	Diseased area > 50%

#### 2.2.2 Pesticide application and leaf collection

**Figure 1:**
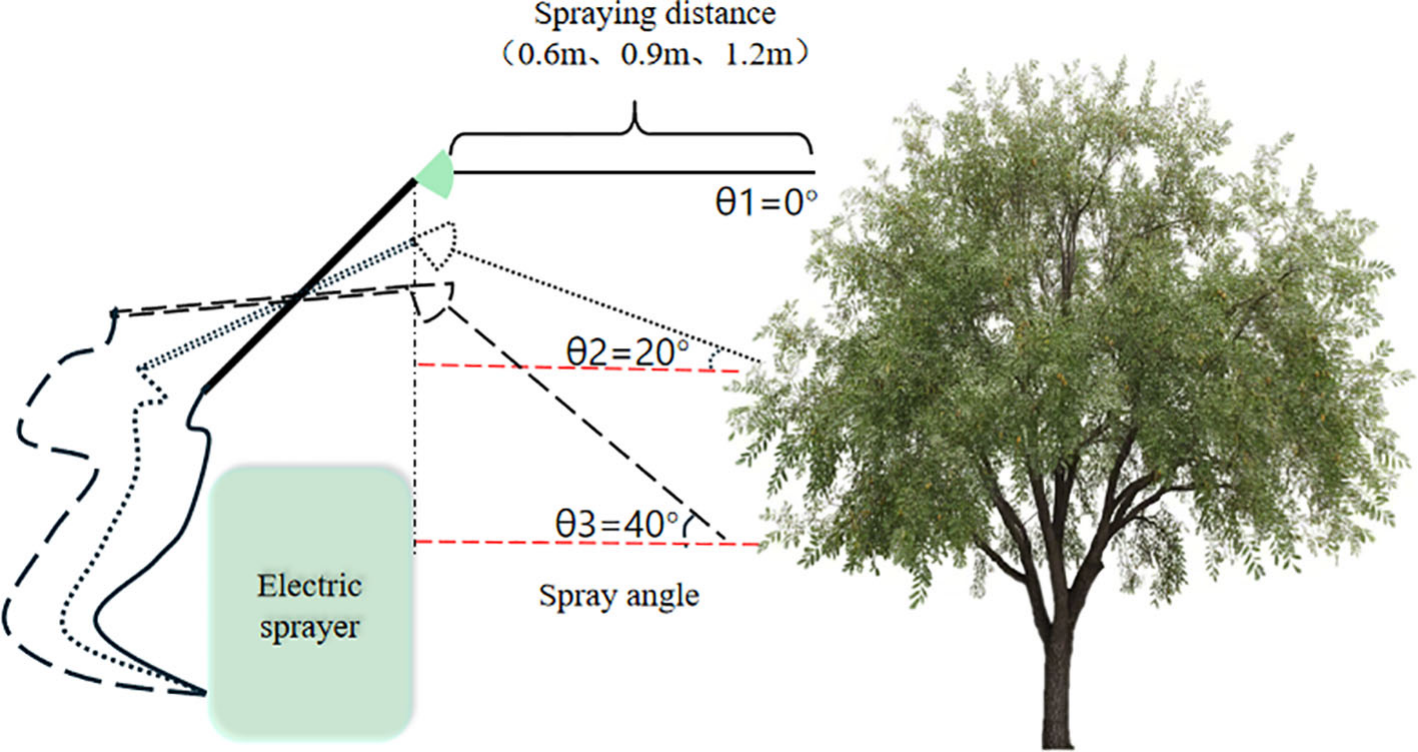
Schematic diagram of chemical spraying treatment.

Leaves with varying degrees of infection in the jujube orchard were tagged, photographed, and numbered (1000 leaves in total were marked). A 20 L electric high-pressure sprayer (Taizhou Yizhou Agricultural Machinery Co., Ltd.) was used to apply the fungicide to leaves of each disease grade using different spraying distances and angles. Different application parameters were set based on spraying distance and angle: spraying distances were set at 0.6 m, 0.9 m, and 1.2 m, and angles were set at 0°, 20°, and 40°. A total of 9 treatments were established: L1 (0.6 m, 0°), L2 (0.6 m, 20°), L3 (0.6 m, 40°), L4 (0.9 m, 0°), L5 (0.9 m, 20°), L6 (0.9 m, 40°), L7 (1.2 m, 0°), L8 (1.2 m, 20°), and L9 (1.2 m, 40°) (**Figure 1**). A control group sprayed with clear water was included. One hundred leaves were sprayed for each treatment. Spraying was conducted once every 7 days, for a total of 3 applications. Leaves from different disease grades and treatments were collected 21 days later.

#### 2.2.3 Measured indicators and methods

The control efficiency after pesticide application was calculated based on the change in the percentage of lesion area (Wang et al., 2025). The malondialdehyde (MDA) content in jujube leaves was determined using the thiobarbituric acid (TBA) method. The soluble sugar (SS) content was determined using the anthrone colorimetric method. The activities of antioxidant enzymes - superoxide dismutase (SOD), peroxidase (POD), and catalase (CAT) - were determined using the nitroblue tetrazolium (NBT) method, guaiacol method, and ultraviolet absorption method, respectively. Phenylalanine ammonia-lyase (PAL) activity was determined using the phenylalanine colorimetric method.

The formula for calculating control efficiency is as follows:


$$\begin{array}{c} \text{Control efficiency }\left(\%\right)\\ =[(\text{Increase in lesion area percentage in control}\\ -\text{Increase in lesion area percentage in treatment})/\\ \text{Increase in lesion area percentage in control}]\times 100 \end{array}$$

### 2.3 Data processing methods

**Figure 2:**
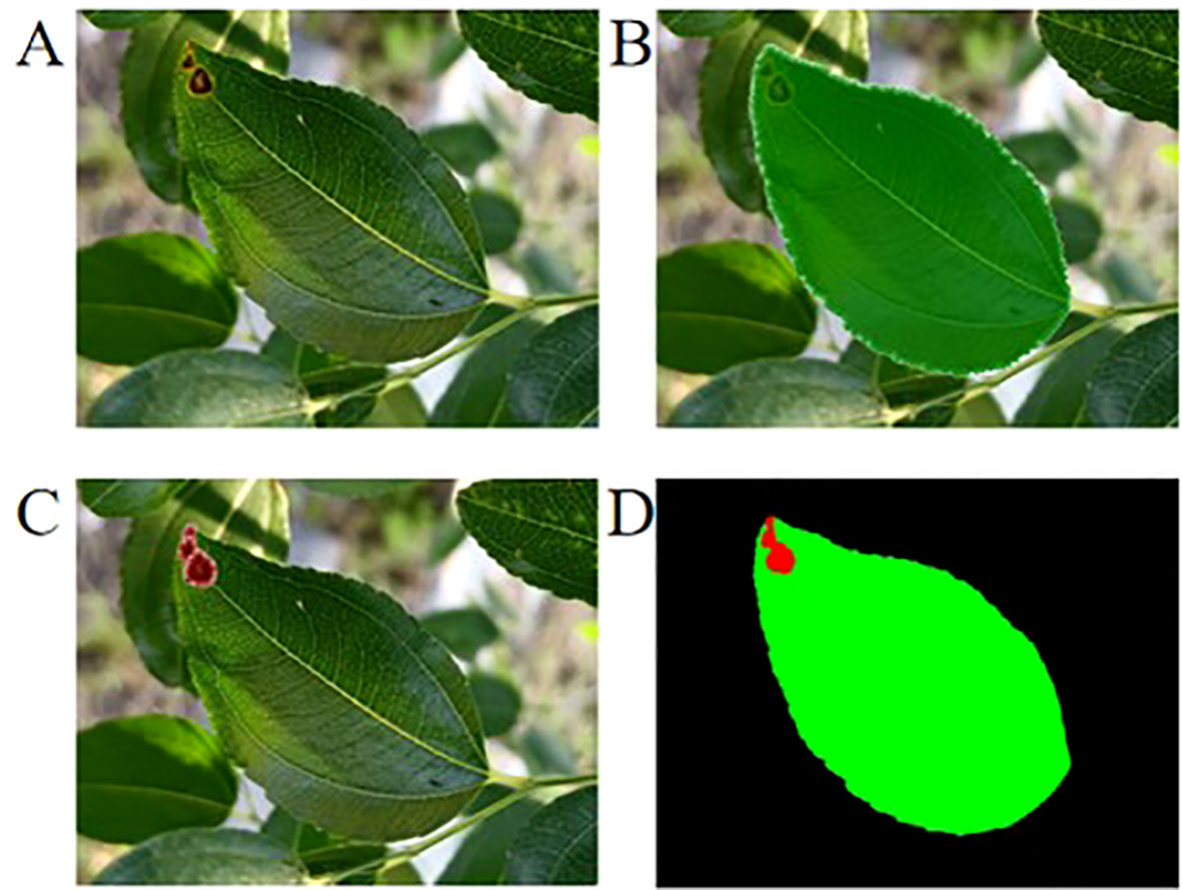
Calculation of lesion area percentage in jujube leaf black spot disease. **(A)** Original field-captured image of jujube leaves; **(B)** Annotated diagram of leaf area; **(C)** Annotated diagram of lesion area; **(D)** Diagram of lesion area calculation results.

Pictures of jujube leaves taken before and after spraying were collected, organized, and then manually annotated using LabelMe (Shu et al., 2023) to mark the leaves and lesions in the images. A Python algorithm was used to calculate the percentage of lesion area (**Figure 2**). The sprayed leaves were classified according to the disease grade criteria. Experimental data were organized using Excel 2016 software. SPSS statistical software was used for one-way analysis of variance (ANOVA) among multiple groups, followed by Duncan’s multiple range test for *post-hoc* comparisons. Origin 2021 was used for chart creation, as well as for significance, correlation, and principal component analyses.

### 2.4 Preliminary development of a science popularization mini-program for jujube leaf black spot disease control

To better apply the hierarchical spraying technology for jujube black leaf spot disease in practical production, we have developed a WeChat mini-program based on existing research findings (Gu, 2025) using WeChat Developer Tools. By combining spraying parameters with the control efficiency against jujube black leaf spot disease at different severity levels, we identified the optimal prevention and treatment parameters for each disease grade. Additionally, we preliminarily established the “Jujube Orchard Manager” WeChat mini-program to popularize the typical symptom characteristics, occurrence patterns, and targeted control measures for grade 1 to 4 jujube black leaf spot disease. This aims to help users more conveniently understand hierarchical disease management and pesticide application.

## 3 Results and analysis

### 3.1 Control efficiency of chemical treatments on jujube leaves with different black spot disease severity grades

To evaluate the control efficiency of the 43% Fluopyram + Tebuconazole SC on jujube leaf black spot disease, the percentage of lesion area was calculated before and after treatment for leaves of different disease severity grades. As shown in **Table 2**, for healthy leaves (Grade 0), significant differences existed among all spraying treatments, indicating that spraying parameters are an important factor affecting the control efficiency.

**Table 2:** Control efficiency in jujube leaves with different disease severity levels of black spot after pesticide application.

Spraying parameters	Control efficiency(%)				
	Grade 0	Grade 1	Grade 2	Grade 3	Grade 4
L1(0.6m, 0°)	94.85 ± 0. 08b	92.60 ± 0. 06d	96.49 ± 0. 02c	97.53 ± 0. 03c	97.74 ± 0. 02d
L2(0.6m, 20°)	78.31 ± 0. 22h	78.60 ± 0. 51i	79.31 ± 0. 08i	99.44 ± 0. 02a	90.74 ± 0. 09h
L3(0.6m, 40°)	91.41 ± 0. 03c	95.19 ± 0. 04c	99.38 ± 0. 03a	96.32 ± 0. 02d	98.44 ± 0. 02c
L4(0.9m, 0°)	97.15 ± 0. 04a	97.43 ± 0. 02b	97.92 ± 0. 05b	98.56 ± 0. 01b	99.09 ± 0. 01b
L5(0.9m, 20°)	79.23 ± 0. 22g	81.76 ± 0. 26h	84.24 ± 0. 43h	84.83 ± 0.40i	84.50 ± 0. 12i
L6(0.9m, 40°)	81.11 ± 0. 21f	80.33 ± 0. 08g	94.41 ± 0. 03d	94.73 ± 0.03e	99.52 ± 0. 01a
L7(1.2m, 0°)	87.90 ± 0. 10d	99.45 ± 0. 04a	92.21 ± 0. 05e	92.85 ± 0. 04f	96.76 ± 0. 02e
L8(1.2m, 20°)	82.81 ± 0. 08e	89.32 ± 0. 09e	89.39 ± 0. 07f	90.70 ± 0. 04g	95.72 ± 0. 01f
L9(1.2m, 40°)	94.85 ± 0. 08b	85.20 ± 0. 11f	86.01 ± 0. 10g	85.86 ± 0. 18h	94.28 ± 0. 06g

Different lowercase letters indicate significant differences between treatments within the same disease severity level (P< 0.05).

For Grade 1 diseased leaves, the control efficiency varied with spraying distance. At spraying angles of 0° and 20°, the control efficiency showed an increasing trend as the spraying distance increased, with the best efficiency observed at 1.2 m, which was significantly higher than at 0.6 m and 0.9 m. At a spraying angle of 40°, the control efficiency initially decreased and then increased with increasing distance, with the best efficiency at 0.6 m. The control efficiency also varied with spraying angle. At a spraying distance of 0.6 m, the control efficiency initially decreased and then increased with increasing spraying angle; the best efficiency was observed at 40°, followed by 0°, and the worst at 20°. At spraying distances of 0.9 m and 1.2 m, the control efficiency decreased as the spraying angle increased.

For healthy leaves (Grade 0), the control efficiency of treatment L4 (spraying distance 0.9 m, angle 0°) was significantly higher than other treatments, with an efficiency of 97.15%. For Grade 1 leaves, treatment L7 (spraying distance 1.2 m, angle 0°) had significantly higher control efficiency than others, at 99.75%. For Grade 2 leaves, treatment L3 (spraying distance 0.6 m, angle 40°) showed significantly higher efficiency, at 99.38%. For Grade 3 leaves, treatment L2 (spraying distance 0.6 m, angle 20°) was significantly superior, with an efficiency of 99.44%. For Grade 4 leaves, treatment L6 (spraying distance 0.9 m, angle 40°) achieved the highest control efficiency, at 99.52%. The results indicate that spraying parameters significantly affected the control efficiency against jujube leaf black spot disease across different severity grades (p< 0.05).

### 3.2 Malondialdehyde content in jujube leaves of different black spot disease grades after chemical treatment

**Figure 3:**
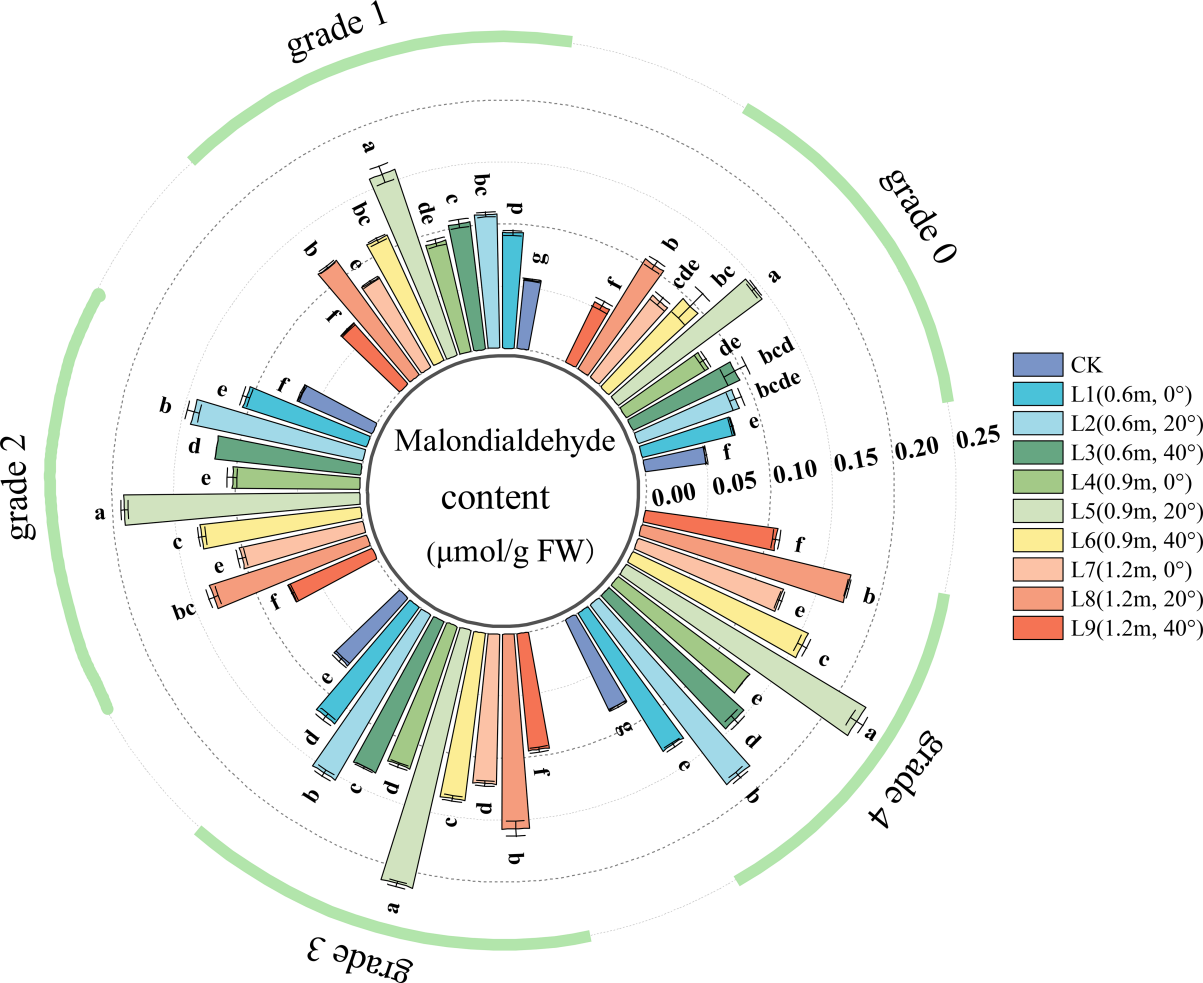
MDA (Malondialdehyde) content in jujube leaves with different disease severity levels of black spot after pesticide application. Different lowercase letters indicate significant differences between treatments within the same disease severity level (P < 0.05). The same below.

The Malondialdehyde (MDA) content reflects the degree of peroxidation of plant cell membranes. A higher MDA content indicates a higher degree of membrane lipid peroxidation and greater damage to the cell membrane. As shown in **Figure 3**, in healthy leaves (Grade 0), all spraying treatments except L9 (spraying distance 1.2 m, angle 40°) significantly increased the leaf MDA content, indicating that the pesticide application caused varying degrees of damage to the leaves.

For Grade 1 diseased leaves, the leaf MDA content varied with spraying distance. At a spraying angle of 0°, there was no significant difference in MDA content among different spraying distances. At a spraying angle of 20°, the leaf MDA content initially increased and then decreased with increasing spraying distance, reaching the highest value at 0.9 m, which was significantly higher than at 0.6 m and 1.2 m (the latter two showed no significant difference). At a spraying angle of 40°, closer spraying distances (0.6 m, 0.9 m) resulted in higher leaf MDA content. The leaf MDA content also varied with spraying angle. At spraying distances of 0.6 m and 0.9 m, the leaf MDA content initially increased and then decreased with increasing spraying angle; the highest MDA content was observed at 20°, followed by 40°, and the lowest at 0°. At a spraying distance of 1.2 m, the leaf MDA content also showed an initial increase followed by a decrease with increasing angle, being highest at 20°, followed by 0°, and lowest at 40°.

In the chemical treatments for Grade 2, Grade 3, and Grade 4 diseased leaves, the effects of spraying angle and distance on leaf MDA content were generally similar to those observed in Grade 1 leaves.

### 3.3 Soluble sugar content in jujube leaves of different black spot disease grades after chemical treatment

**Figure 4:**
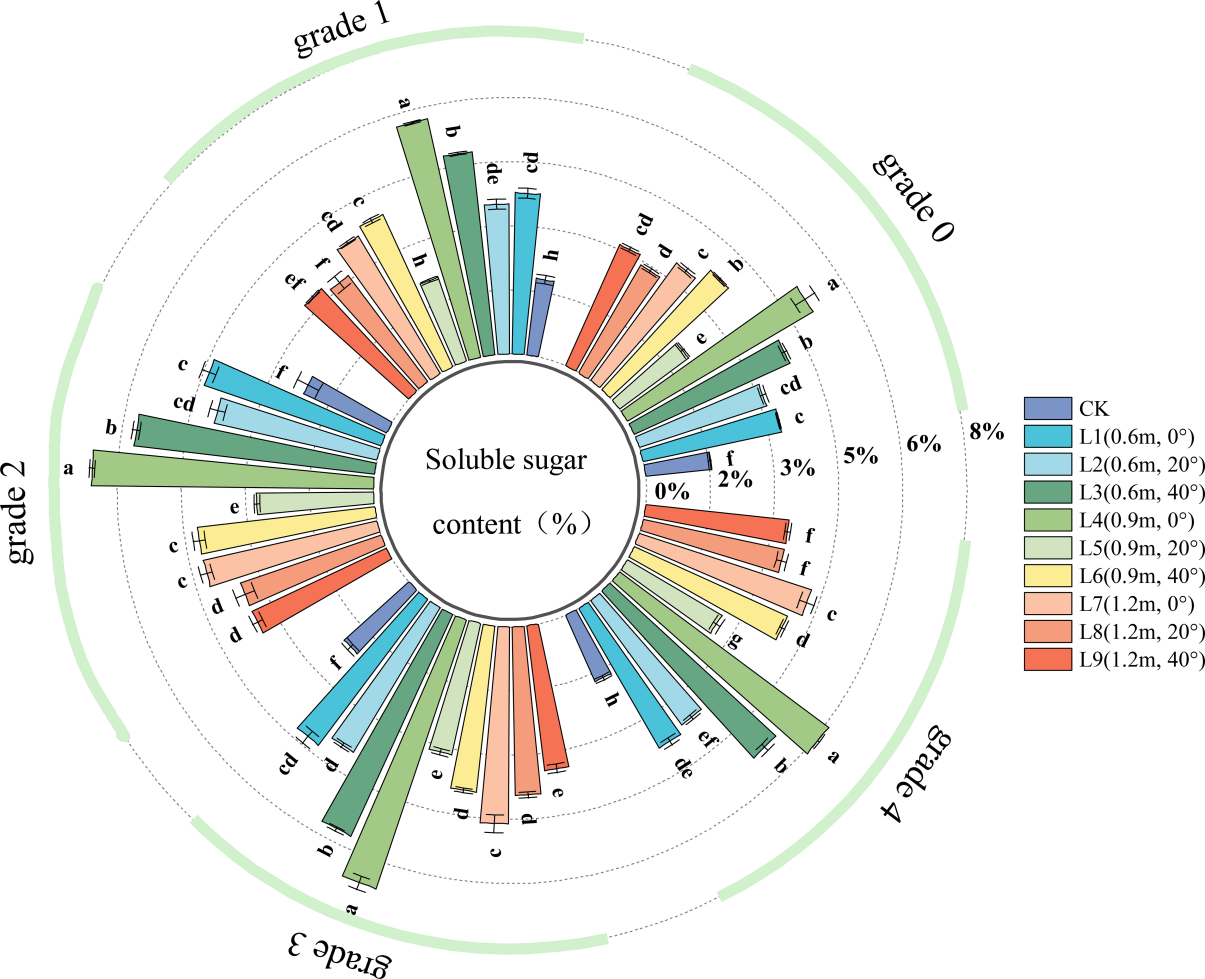
SS (Soluble Sugar) content in jujube leaves with different disease severity levels of black spot after fungicide application.

The SS content is an important indicator of plant cell energy reserves and metabolic activity. A higher soluble sugar content indicates richer energy reserves and more active metabolic processes in plant cells. As shown in **Figure 4**, in healthy leaves (Grade 0), all spraying treatments significantly increased the leaf SS content, indicating that the pesticide application induced the leaves to enhance stress resistance by accumulating osmoregulatory substances.

For Grade 1 diseased leaves, the leaf SS content varied with spraying distance. At a spraying angle of 0°, the leaf SS content was highest at the 0.9 m distance, significantly higher than at 0.6 m and 1.2 m (the latter two showed no significant difference). At a spraying angle of 20°, the leaf SS content initially decreased and then increased with increasing spraying distance, being highest at 0.6 m, significantly higher than at 0.9 m and 1.2 m. At a spraying angle of 40°, closer spraying distances (0.6 m, 0.9 m) resulted in higher leaf SS content. The leaf SS content also varied with spraying angle. As the spraying angle increased, the leaf SS content initially decreased and then increased; the SS content was lowest at a 20° angle. At spraying distances of 0.9 m and 1.2 m, the SS content was highest at a 0° angle, while at a 0.6 m distance, the SS content was highest at a 40° angle.

The SS content peaked at disease Grade 2 and Grade 3. In the chemical treatments for Grade 2, Grade 3, and Grade 4 diseased leaves, the effects of spraying angle and distance on leaf SS content were generally similar to those observed in Grade 1 leaves. After chemical treatment, within the same disease grade, the SS content in treatment L4 (spraying distance 0.9 m, angle 0°) was significantly higher than in other treatments. Compared to Grade 0, the SS content in L4 increased relatively by 13.25%, 31.13%, 31.79%, and 21.20% for disease Grades 1, 2, 3, and 4, respectively.

### 3.4 Superoxide dismutase activity in jujube leaves of different black spot disease grades after chemical treatment

**Figure 5:**
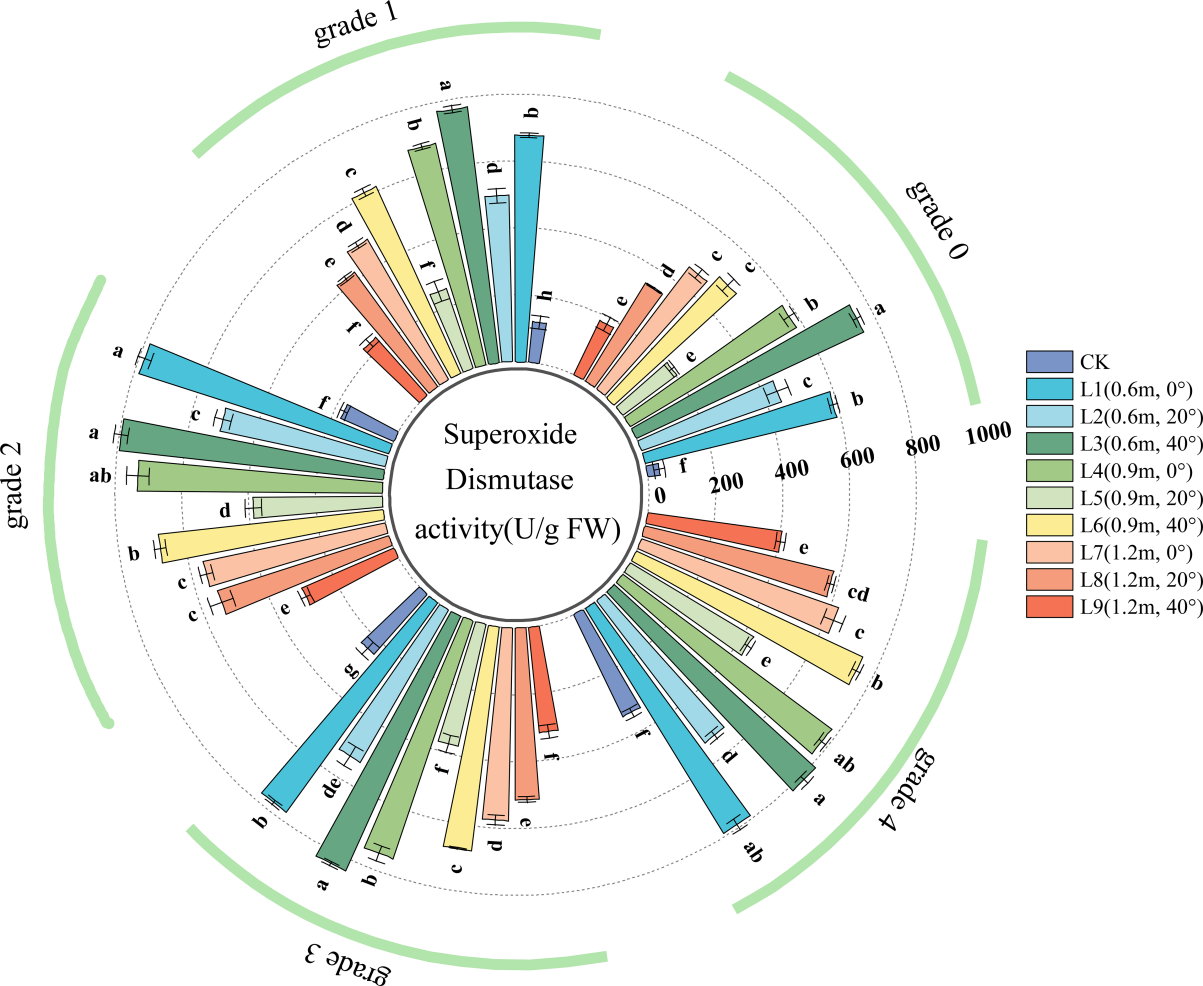
SOD (Superoxide Dismutase) activity in jujube leaves with different disease severity levels of black spot after fungicide application.

SOD activity is an important indicator of a plant’s ability to scavenge reactive oxygen species (ROS). Higher SOD activity indicates a stronger antioxidant defense system and a more significant resistance to oxidative damage. As shown in **Figure 5**, in healthy leaves (Grade 0), all spraying treatments significantly increased leaf SOD activity, indicating that the pesticide application activated the leaf’s antioxidant protection mechanism, effectively mitigating oxidative damage to cellular structures.

For Grade 1 diseased leaves, leaf SOD activity varied with spraying distance. At a spraying angle of 0°, SOD activity was lowest at 1.2 m, significantly lower than at 0.6 m and 0.9 m (the latter two showed no significant difference). At a spraying angle of 20°, SOD activity initially decreased and then increased with increasing spraying distance; SOD activity was highest at a spraying distance of 0.6 m, followed by 1.2 m, and lowest at 0.9 m. Leaf SOD activity also varied with spraying angle. At spraying distances of 0.6 m and 0.9 m, SOD activity initially decreased and then increased with increasing spraying angle, reaching its lowest point at 20°. At a spraying distance of 1.2 m, SOD activity decreased as the spraying angle increased.

In the chemical treatments for Grade 2, Grade 3, and Grade 4 diseased leaves, the effects of spraying angle and distance on leaf SOD activity were generally similar to those observed in Grade 1 leaves. After treatment across disease Grades 0 ~ 4, SOD activity in jujube leaves from treatment L3 (spraying distance 0.6 m, angle 40°) was significantly higher than in other treatments, while treatment L9 (spraying distance 1.2 m, angle 40°) resulted in the lowest SOD activity.

### 3.5 Peroxidase activity in jujube leaves of different black spot disease grades after chemical treatment

**Figure 6:**
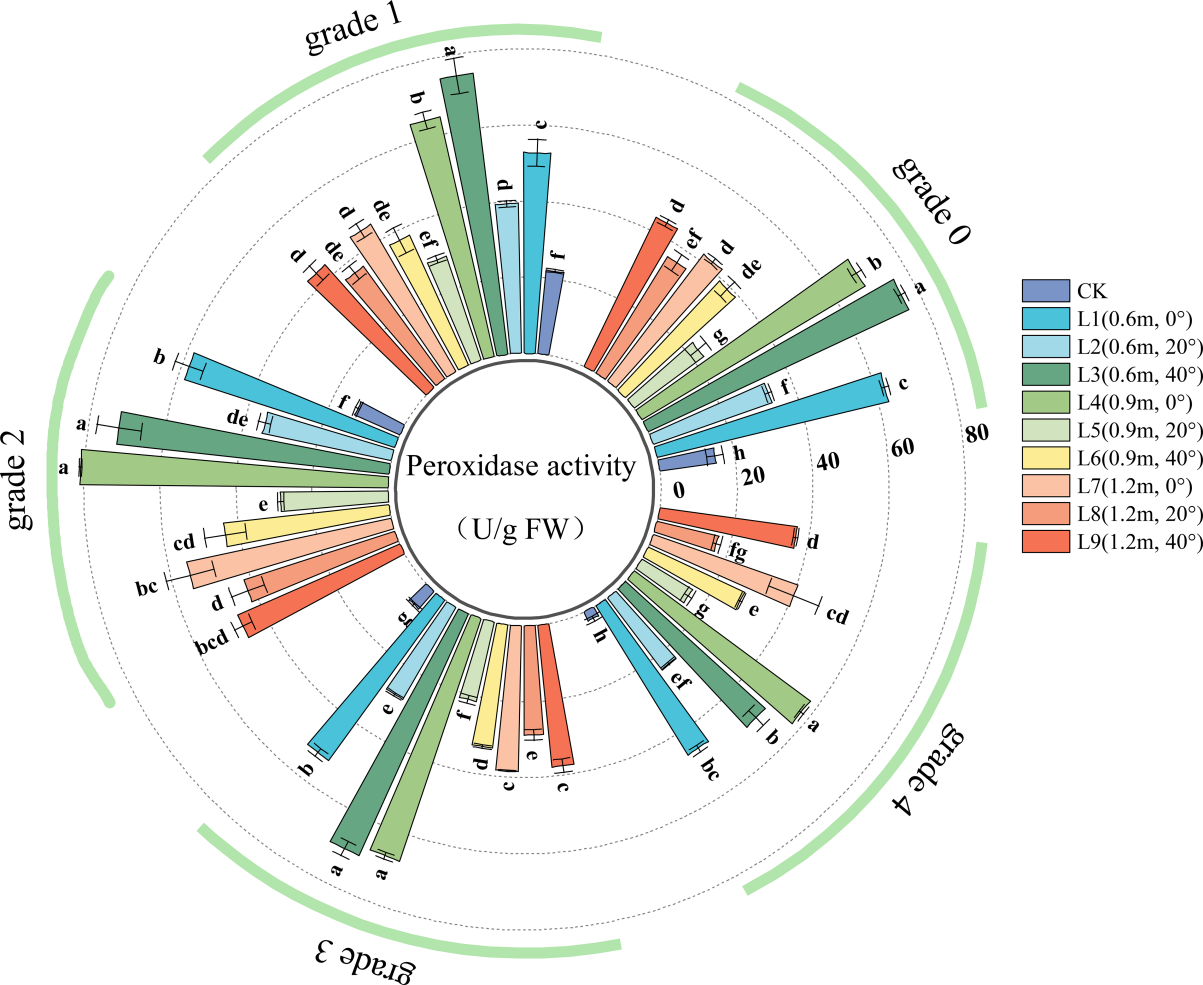
POD (Peroxidase) activity in jujube leaves with different disease severity levels of black spot after fungicide application.

POD activity is a key indicator of a plant’s ability to scavenge ROS such as hydrogen peroxide. Higher POD activity indicates greater efficiency in clearing oxidative stress and a more robust protective mechanism for the cell membrane system. As shown in **Figure 6**, in healthy leaves (Grade 0), all spraying treatments significantly increased leaf POD activity, indicating that the pesticide application enhanced the leaf’s antioxidant defense capacity, maintaining the stability of the cell membrane structure by efficiently clearing ROS like hydrogen peroxide.

For Grade 1 diseased leaves, leaf POD activity varied with spraying distance. At a spraying angle of 0°, POD activity initially increased and then decreased with increasing spraying distance. At spraying angles of 20° and 40°, POD activity initially decreased and then increased with increasing distance; POD activity was highest at 0.6 m, significantly higher than at 0.9 m and 1.2 m (the latter two showed no significant difference). Leaf POD activity also varied with spraying angle. As the spraying angle increased, POD activity initially decreased and then increased. At a spraying distance of 0.6 m, POD activity was highest at 40°, followed by 0°, and lowest at 20°. At a distance of 0.9 m, POD activity was highest at 0°, followed by 40°, and lowest at 20°. At a distance of 1.2 m, POD activity at 20° was significantly lower than at 0° and 40° (the latter two showed no significant difference).

In the chemical treatments for Grade 2, Grade 3, and Grade 4 diseased leaves, the effects of spraying angle and distance on leaf POD activity were generally similar to those observed in Grade 1 leaves. After treatment, within the same disease grade, POD activity in treatments L3 (spraying distance 0.6 m, angle 40°) and L4 (spraying distance 0.9 m, angle 0°) was significantly higher than in other treatments; POD activity was lowest in treatments L2 (0.6 m, 20°), L5 (0.9 m, 20°), and L8 (1.2 m, 20°).

### 3.6 Catalase activity in jujube leaves of different black spot disease grades after chemical treatment

**Figure 7:**
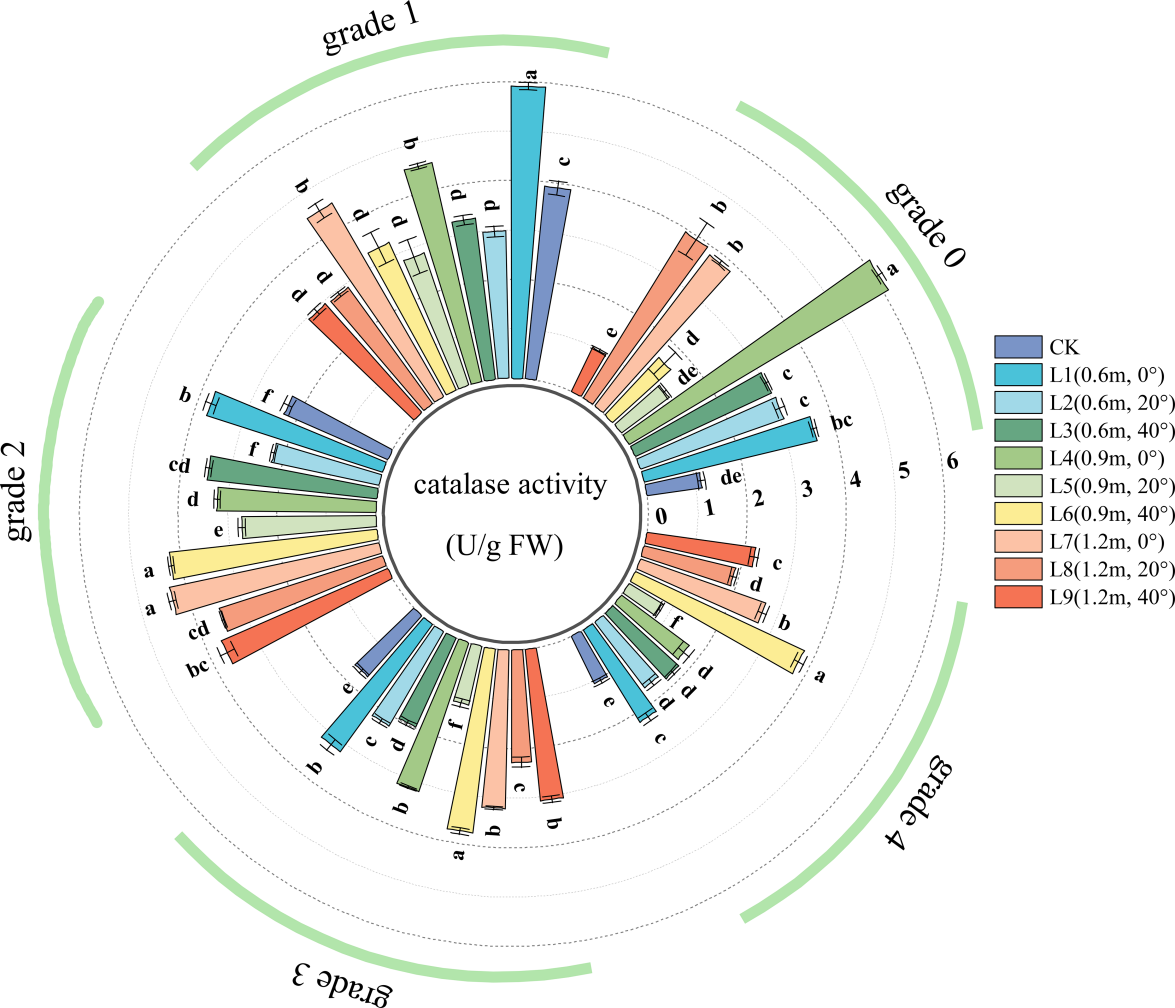
CAT (Catalase) activity in jujube leaves with different disease severity levels of black spot after fungicide application.

CAT activity is an important marker of a plant’s ability to decompose hydrogen peroxide. Higher CAT activity indicates greater efficiency in breaking down ROS and a more prominent capacity for repairing cellular oxidative damage. As shown in **Figure 7**, in healthy leaves (Grade 0), except for treatments L5 (0.9 m, 20°), L6 (0.9 m, 40°), and L9 (1.2 m, 40°), all spraying treatments significantly increased leaf CAT activity. This indicates that the pesticide application enhanced the leaves’ ability to scavenge hydrogen peroxide, effectively protecting the cell membrane system from oxidative damage by maintaining ROS metabolic balance.

For Grade 1 diseased leaves, leaf CAT activity varied with spraying distance. At spraying angles of 0° and 40°, CAT activity decreased with increasing spraying distance; it was highest at 0.6 m, with no significant difference between 0.9 m and 1.2 m. At a spraying angle of 20°, there was no significant difference in CAT activity among different spraying distances. Leaf CAT activity also varied with spraying angle. As the spraying angle increased, CAT activity at distances of 0.6 m and 0.9 m initially decreased and then increased; it was highest at 0°, followed by 40°, and lowest at 20°. At a spraying distance of 1.2 m, CAT activity showed a decreasing trend with increasing angle.

In the chemical treatments for Grade 2, Grade 3, and Grade 4 diseased leaves, the effects of spraying angle and distance on leaf CAT activity were generally similar to those observed in Grade 1 leaves. CAT activity showed a linear increasing trend from disease Grade 0 to Grade 1, and a decreasing trend from Grade 2 to Grade 4. For disease Grades 1~4, CAT activity was highest in leaves from treatment L1 (0.6 m, 0°). As the disease grade increased, the decline in CAT activity was smallest in treatment L6 (0.9 m, 40°).

### 3.7 Phenylalanine ammonia-lyase activity in jujube leaves of different black spot disease grades after chemical treatment

**Figure 8:**
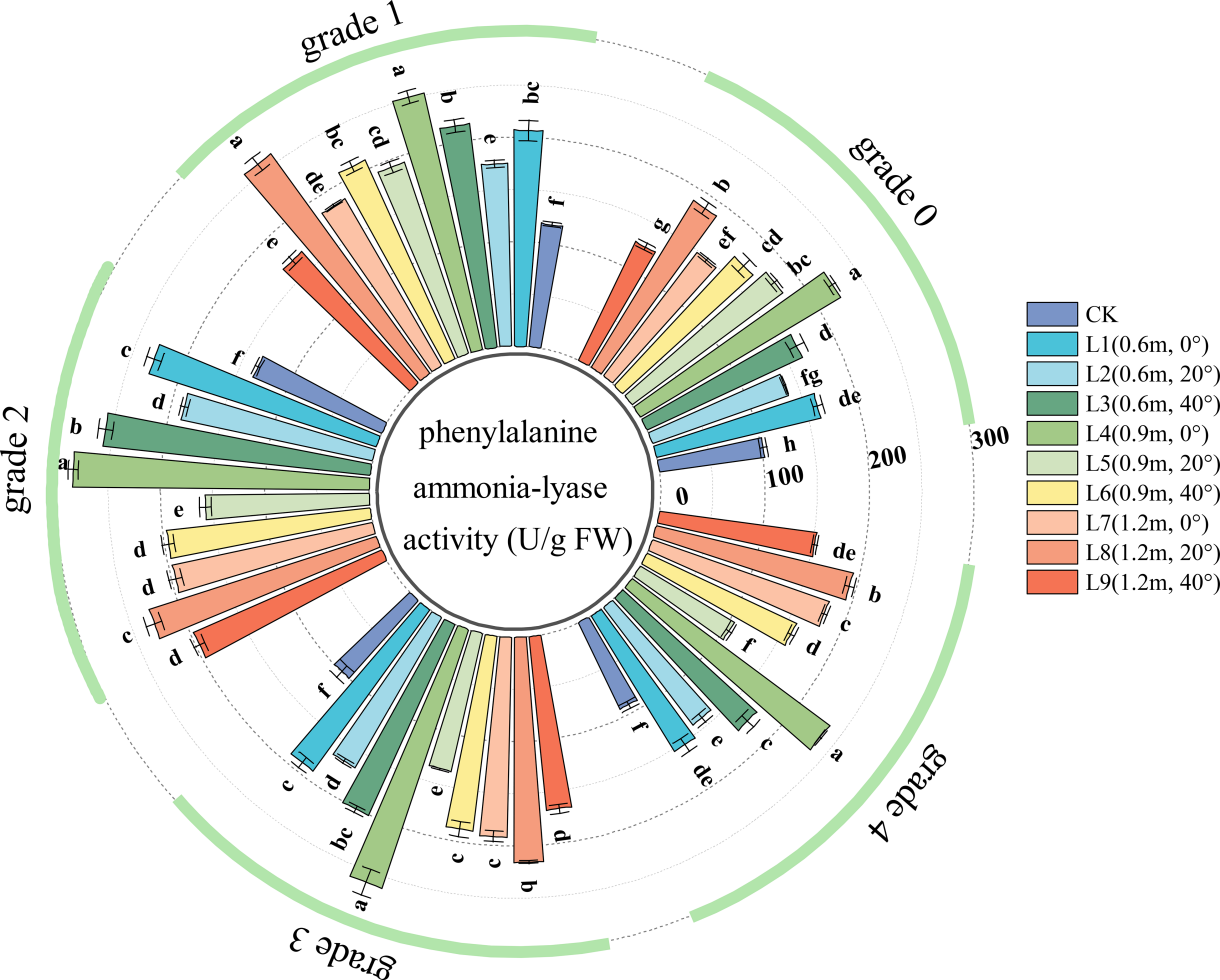
PAL (Phenylalanine Ammonia-Lyase) activity in jujube leaves with different disease severity levels of black spot after fungicide application.

PAL activity is a key indicator of the intensity of the phenylpropanoid metabolic pathway in plants. Higher PAL activity indicates a stronger capacity to synthesize secondary metabolites such as phenolics and lignin, and a more prominent active defense capability against pathogen infection or environmental stress. As shown in **Figure 8**, in healthy leaves (Grade 0), all spraying treatments significantly increased leaf PAL activity, indicating that the pesticide application activated the phenylpropanoid pathway, enhancing the leaf’s defense capability against biotic and abiotic stress by accelerating the synthesis of phenolics and lignin.

For Grade 1 diseased leaves, leaf PAL activity varied with spraying distance. As the spraying distance increased, PAL activity initially increased and then decreased at a 0° angle, showed an increasing trend at a 20° angle, and a decreasing trend at a 40° angle. Leaf PAL activity also varied with spraying angle. As the spraying angle increased, PAL activity at distances of 0.6 m and 0.9 m initially decreased and then increased while at a spraying distance of 1.2 m, PAL activity showed a decreasing trend.

In the chemical treatments for Grade 2, Grade 3, and Grade 4 diseased leaves, the effects of spraying angle and distance on leaf PAL activity were generally similar to those observed in Grade 1 leaves. After treatment, PAL activity in treatment L4 (0.9 m, 0°) was significantly higher than in other treatments; PAL activity was also prominent in treatments L3 (0.6 m, 40°), L6 (0.9 m, 40°), and L8 (1.2 m, 20°); PAL activity was lowest in treatments L2 (0.6 m, 20°), L5 (0.9 m, 20°), and L9 (1.2 m, 40°).

In summary, the results indicate that as the disease grade increased, MDA content and SOD activity showed an increasing trend, while SS content, POD activity, CAT activity, and PAL activity initially increased and then decreased. Within the same disease grade, as the spraying angle increased, MDA content (for disease grades 1~4), SS content, SOD activity (at spraying distances of 0.6 m and 0.9 m), POD activity, CAT activity, and PAL activity generally showed an initial decrease followed by an increase. As the spraying distance increased, MDA content, SS content, SOD activity, POD activity, CAT activity, and PAL activity, at a spraying angle of 0°, initially increased and then decreased; at a spraying angle of 20°, they initially decreased and then increased; and at a spraying angle of 40°, they generally showed a decreasing trend.

### 3.8 Correlation and principal component analysis

**Figure 9:**
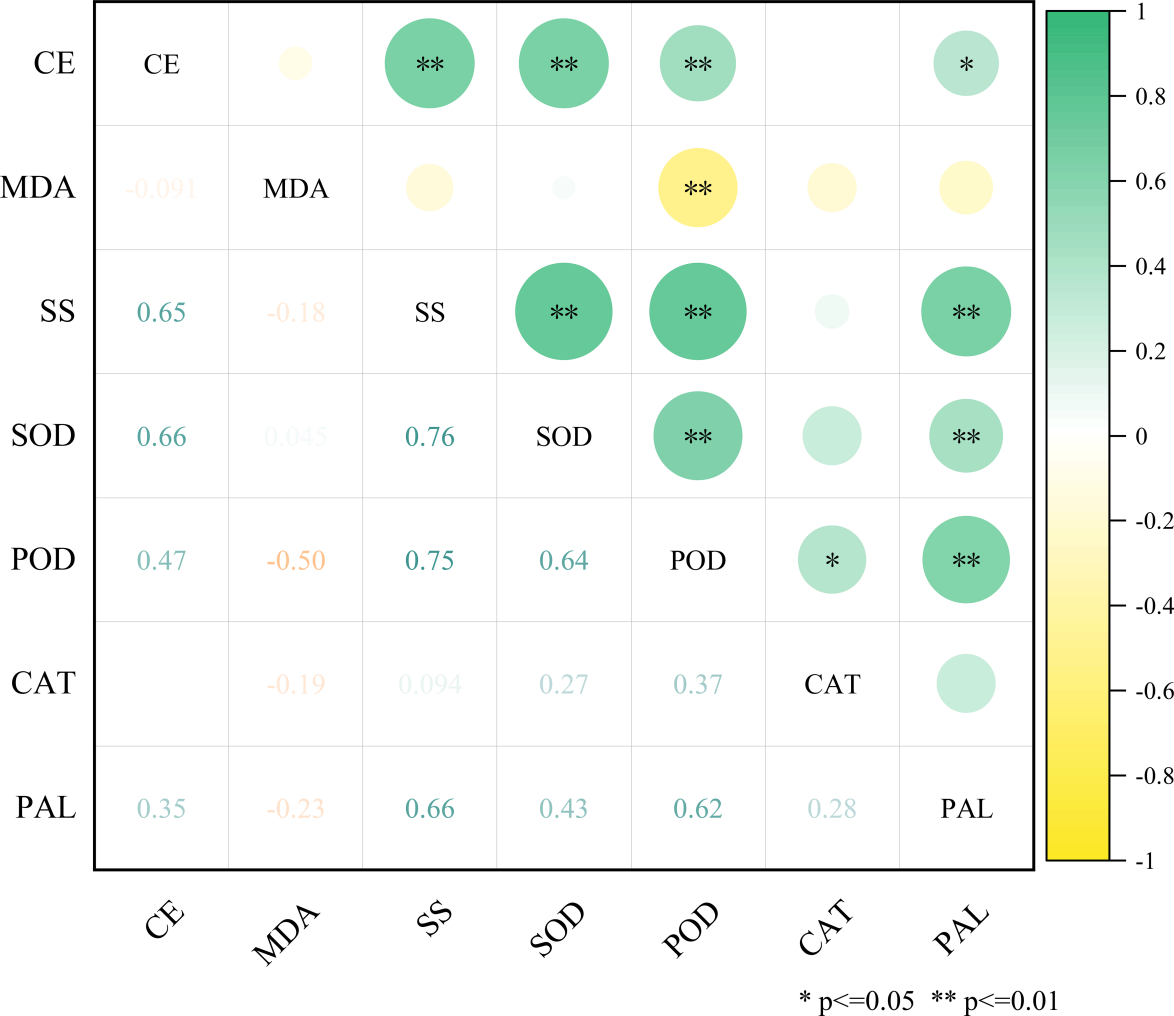
Correlation between control efficiency and physiological indicators. CE, Control efficiency; MDA, Malondialdehyde content; SS, Soluble sugar content; SOD, Superoxide dismutase activity; POD, Peroxidase activity; CAT, Catalase activity; PAL, Phenylalanine ammonia-lyase activity. The color intensity and circle size are proportional to the value of each correlation coefficient. Green represents a positive correlation, and yellow represents a negative correlation. * represents a significant correlation at the 0.05 level; ** indicates a significant correlation at 0.01 level.

Correlation analysis is a multivariate statistical method that measures the degree of association between variables, using statistical indicators to reveal internal data relationships, thereby identifying key relationships and patterns. Correlation analysis showed (**Figure 9**) that under different spraying parameters, the correlations between control efficiency and physiological indicators varied significantly. Control efficiency showed a highly significant positive correlation (P< 0.05) with SS content, SOD activity, and POD activity, and a significant positive correlation (P< 0.05) with PAL activity. There was almost no correlation with CAT activity or MDA content. This indicates that stronger disease resistance, stress tolerance, and antioxidant capacity in plants after pesticide application are more conducive to improving disease control efficiency.

**Figure 10:**
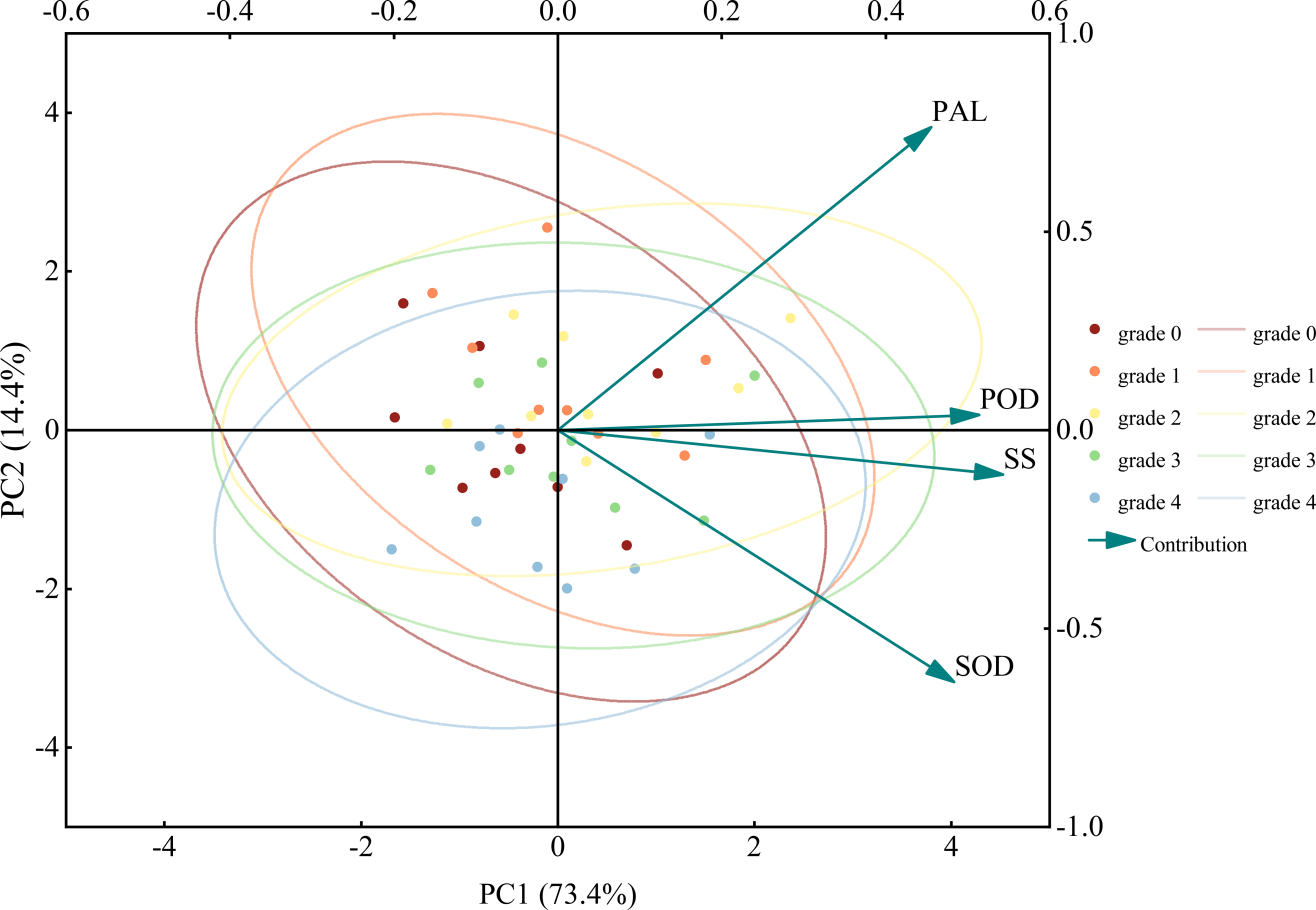
PCA biplot of physiological indices in jujube leaves with different severity levels of black spot after disease control. Circles indicate 95% confidence intervals, and arrows represent loading vectors.

PCA is a multivariate statistical method that reduces data dimensionality by transforming multiple variables into a few comprehensive variables through linear transformation, using simplified data to reflect the original dataset. PCA was performed on the four indicator parameters that showed significant correlation with control efficiency. The results showed that when the characteristic value was greater than 1, there were two principal components (PC1, PC2), with their cumulative contribution variance summing to 87.8%. Among them, the contribution rates of PC1 and PC2 were 73.4% and 14.4%, respectively (**Table 3**). **Figure 10** reflects the magnitude of the loadings of each physiological indicator in its principal components. Among them, SOD and POD contributed more to PC1, while PAL contributed more to PC2.

**Table 3:** Eigenvalues and contribution rates of each principal component.

Principal component number	Eigenvalue	Percentage of variance(%)	Cumulative(%)
1	2.93711	73.42774	73.42774
2	0.57443	14.36068	87.78842
3	0.30922	7.73038	95.5188
4	0.17925	4.4812	100

### 3.9 Preliminary development of the WeChat mini-program

**Figure 11:**
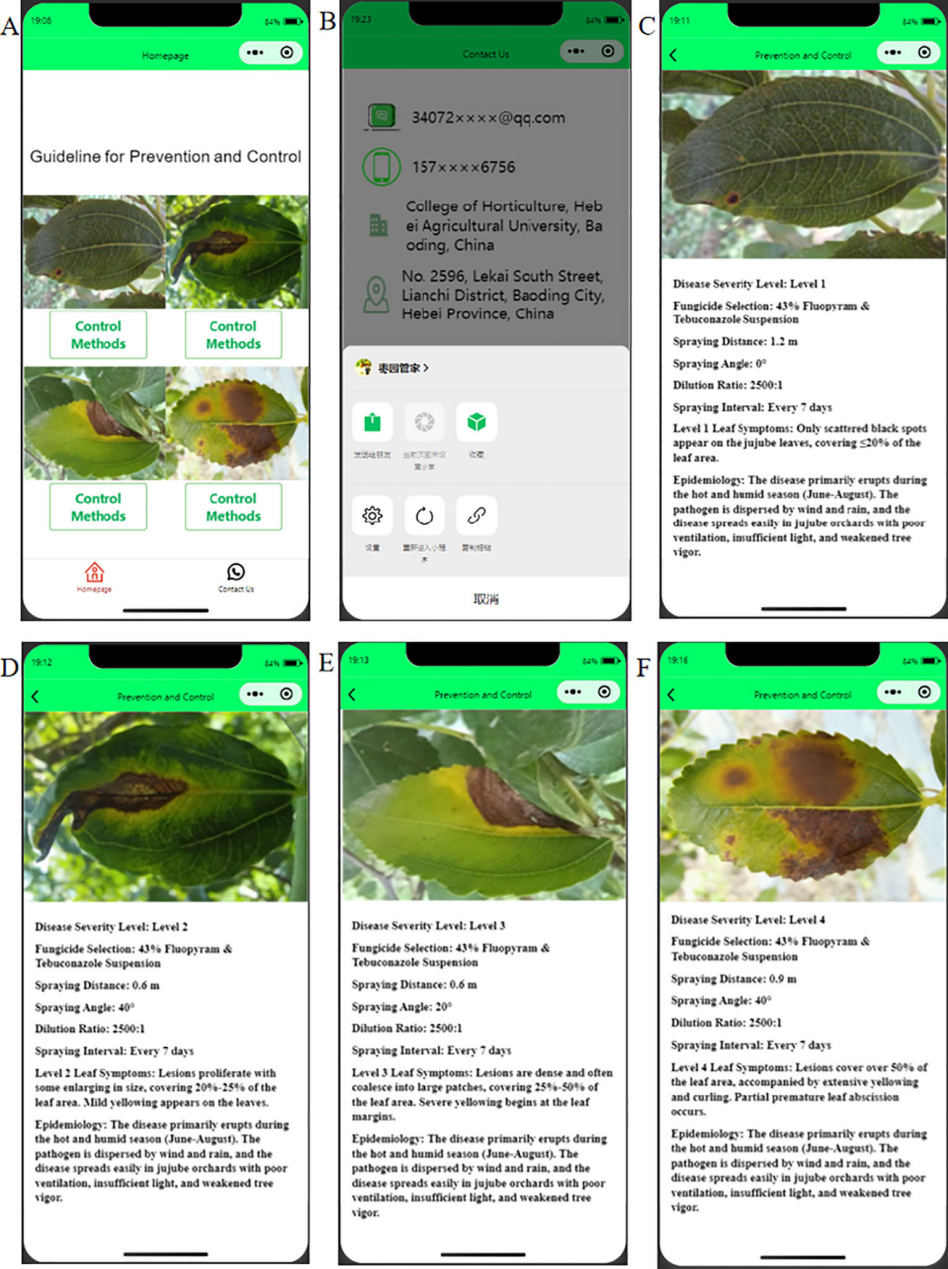
The "Jujube Orchard Manager" Popular Science Mini Program. **(A)** Homepage; **(B)** Quick Contact; **(C–F)** Details Page.

“Jujube Orchard Manager” is a science popularization mini-program built based on WeChat Developer Tools, targeting farmer groups (**Figure 11**). The mini-program’s homepage allows users to compare typical symptom images of jujube leaf black spot disease for specific severity grades. By clicking the “Control Methods” button, users can navigate to the details page to obtain information on the infection environment characteristics and pesticide application guidance corresponding to that disease grade. Additionally, the mini-program features a “Contact Us” quick access entry, facilitating communication between users and the development team. The “Jujube Orchard Manager” mini-program provides users in need with a convenient channel for information access and professional technical support, effectively promoting the dissemination and application of knowledge on graded precision control of jujube tree diseases.

## 4 Discussion

### 4.1 Control efficiency of spraying parameters on jujube leaves with different black spot disease grades

Pesticide application is one of the primary methods for field control. To enhance the control efficiency of pesticides, researchers have conducted extensive studies. The effects of application methods, pesticide dosage, and distribution methods on control efficiency have been widely investigated (Gu et al., 2018, 2022). Zhang et al. (2025b) developed a variable-rate spraying system that synergizes disease severity with canopy volume measurement to achieve adaptive pesticide dose optimization. Wu et al. (2025) significantly increased droplet deposition within the tea plant canopy by optimizing UAV parameters. Numerous studies have demonstrated that optimizing spraying parameters can precisely regulate pesticide distribution and dosage, enhancing targeted control efficiency (Anoumaa et al., 2024; Liu et al., 2025). This study found that, compared to the control, pesticide application significantly delayed lesion spread, and different spraying parameters resulted in significant differences in control efficiency, indicating that the selection of spraying parameters is crucial for maximizing the preventive and control effect of the pesticide.

For healthy leaves (Grade 0), application at a distance of 0.9 m and an angle of 0° resulted in the least lesion spread, with a control efficiency of 97.15%. Therefore, these parameters are inferred to be the optimal for preventing disease occurrence. For diseased leaves of Grades 1~4, the best control efficacies were 99.45%, 99.37%, 99.44%, and 99.52%, respectively. In contrast, the previously reported highest field efficiency of 40% prothioconazole-tebuconazole SC against ginseng black spot disease was 86.50% (Yu et al., 2025). This indicates that the 43% Fluopyram + Tebuconazole SC possesses strong inhibitory activity against jujube leaf black spot disease.

Simultaneously, the study found that for Grade 1 diseased leaves, application at a distance of 1.2 m and an angle of 0° resulted in the least lesion spread. For Grade 2 leaves, a distance of 0.6 m and angle of 40° were best; for Grade 3, 0.6 m and 20°; and for Grade 4, 0.9 m and 40°. This suggests that the coverage, penetration, or deposition efficiency of spray droplets may vary with disease severity. Appropriate spraying distance and angle reduce pesticide drift, while inclination improves coverage on both sides of the leaf, enhancing contact efficiency (Liao et al., 2015; Dong et al., 2025). Precise matching of spraying parameters is key to maximizing pesticide efficiency, collaborating with the operation of precise variable-rate spraying systems (Luo et al., 2025), to optimize control effects for jujube leaves with different black spot disease grades.

### 4.2 Physiological effects of spraying parameters on jujube leaves with different black spot disease grades

The 43% fluoxastrobin + tebuconazole suspension concentrate is composed of 18% fluoxastrobin and 25% tebuconazole, exhibiting a unique synergistic mechanism of action. Fluoxastrobin is a systemic fluorine-containing fungicide that effectively controls pathogens resistant to sterol inhibitors, phenylamides, benzimidazoles, and other existing fungicide classes. It primarily inhibits electron transfer in the cytochrome bc1 complex of the fungal mitochondrial respiratory chain, thereby disrupting energy synthesis. Tebuconazole, on the other hand, inhibits ergosterol biosynthesis, leading to the disruption of cell membrane structure. The combination of these two agents enables multi-target disease control (Yan et al., 2024). This formulation provides high efficacy in both protection and treatment during the early stages of fungal infection and mycelial growth. It is rapidly absorbed through plant leaves and roots, demonstrating significant effectiveness against fungal diseases such as rust, powdery mildew, and anthracnose. It acts quickly, offers long-lasting effects, and is safe for crops. Additionally, fluoxastrobin positively influences plant health by delaying senescence, reducing ethylene production, enhancing carbon assimilation efficiency and water use efficiency, thereby improving leaf color and grain filling (Hua, 2020). Appropriate pesticide concentrations help promote physiological activities in plants, increase enzyme activity and crop quality, enhance resistance, support healthy growth, and improve yield.

**Figure 12:**
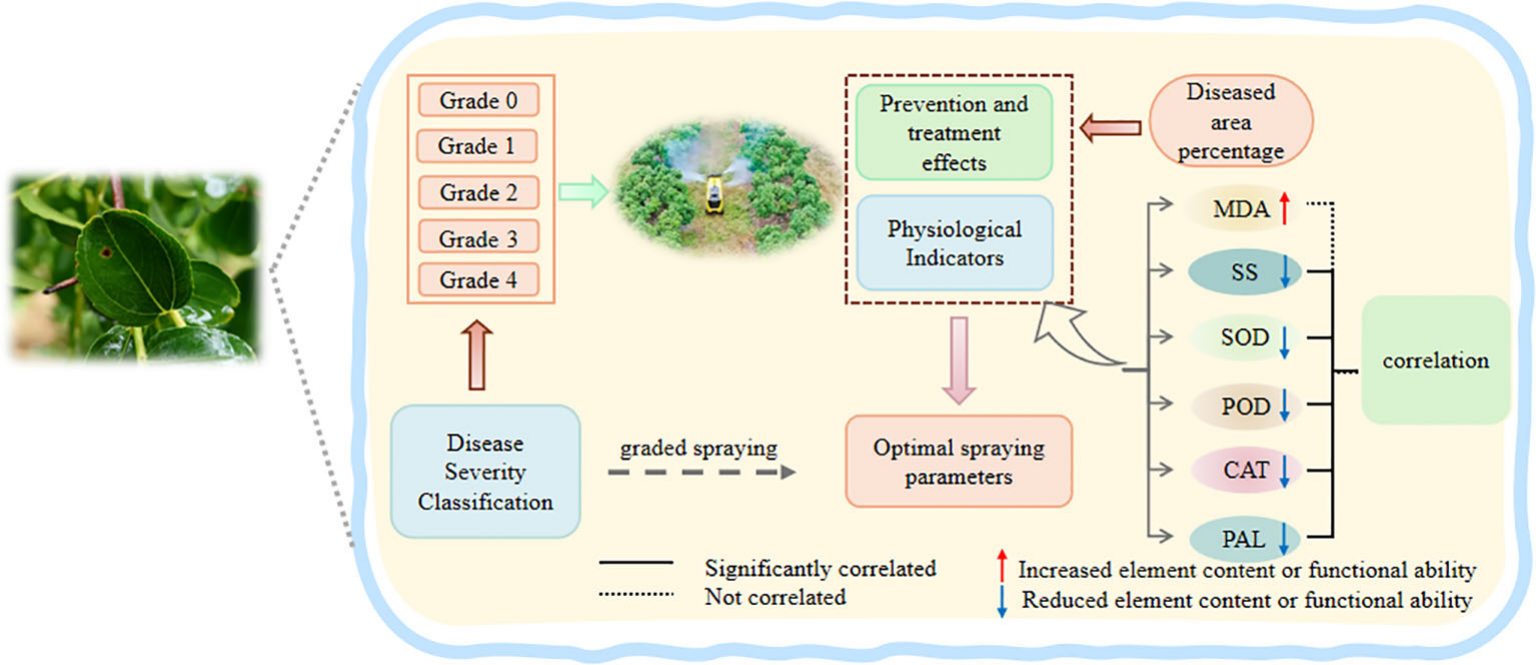
Schematic diagram of the study on effects of spray parameters on jujube leaves with different black spot disease severity levels.

Pesticides dose-dependently regulate antioxidant enzyme activity, reduce membrane lipid peroxidation, and activate sugar metabolism and the phenylpropanoid pathway, thereby synergistically enhancing plant disease resistance (Gill and Tuteja, 2010; Köseoğlu and Doğru, 2025). However, excessive application may inhibit enzyme activity, induce oxidative damage and metabolic imbalance, and reduce plant health (Kakoki et al., 2019). This study investigated the effects of different spraying distances and angles on MDA content, SS content, and the activities of SOD, POD, CAT, and PAL in jujube leaves of different black spot disease grades. The aim was to optimize spraying distance and angle for precise application, reduce leaf damage, and achieve graded disease control with reduced pesticide use and increased efficiency (**Figure 12**). This study found that spraying 43% flufenacet · tebuconazole suspension significantly enhanced the activities of antioxidant enzymes such as SOD, POD, CAT, and PAL in the leaves. This may be attributed to the agent alleviating oxidative damage by strengthening the plant’s antioxidant defense system. By inducing reactive oxygen species (ROS) signals, the agent systematically activated the plant’s “defense alert state.” This not only enhanced the crop’s basic stress resistance capacity by clearing reactive oxygen species and combating adverse stress but also improved its specific disease resistance against subsequent pathogen invasions through strengthened cell walls and accumulation of antimicrobial substances. This result is consistent with the study by Ye et al. (2024), which indicated that increasing the activity of related antioxidant enzymes in plants helps enhance resistance to leaf spot disease.The study further confirms that pesticide application with different parameters significantly affected leaf MDA content, SS content, and SOD, POD, CAT, and PAL activities, indicating that spraying parameters might be key factors regulating physiological and biochemical processes related to plant stress resistance (Yuan et al., 2025). This provides an important theoretical basis for optimizing application strategies to enhance plant self-defense capabilities and reduce phytotoxicity.

Concurrently, the study found that the control efficacy was significantly and positively correlated with the contents of SS and the activities of SOD, POD, and PAL (P< 0.05), while it showed little to no correlation with CAT activity or MDA content. SS content and PAL activity reached their maximum in the treatment with a spraying distance of 0.9 m and angle of 0°. SOD and POD activities were also relatively high in this treatment. Treatments with spraying distances of 0.6 m and 0.9 m at a 40° angle performed notably well in terms of SS content and PAL activity. These results suggest that the treatment at 0.9 m and 0° likely enhanced the control efficiency against jujube leaf black spot disease by strengthening the antioxidant system and disease resistance metabolic pathways (Han et al., 2024; Xu et al., 2024) (Mohammadhadi et al., 2024). The treatments at 0.6 m and 0.9 m with a 40° angle might effectively inhibit the occurrence and development of jujube leaf black spot disease by synergistically inducing the phenylpropanoid pathway and the sterol glycolalkaloid metabolic pathway, thereby enhancing the physical barrier function of the plant cell wall and the accumulation of endogenous antimicrobial substances (Bai et al., 2025; Zhou et al., 2025).

Shi et al. (2025) found that the occurrence of early watercore in apples was closely related to an imbalance in reactive oxygen species metabolism and decreased antioxidant enzyme activities. This study found that after treatment, SS content, POD and PAL activities in jujube leaves initially increased and then decreased with increasing disease grade, peaking at Grade 2, while CAT activity peaked at Grade 1. Yao et al. (2021) found that SOD, POD, and CAT activities increased in mildly watercored pineapple fruits compared to controls, but in moderately watercored fruits, SOD and POD activities decreased significantly while CAT activity increased. This differs from the findings for Grades 2~3 in this study, possibly due to differences in pathogen populations, ecological environments, and climatic factors. Analyzing the physiological effects of spraying parameters on jujube leaves with different black spot disease grades indicates that plant disease resistance has a critical point. Moderate stress can enhance defense mechanisms, but excessive stress inhibits enzyme activity, disrupts sugar metabolism, weakens the synthesis of disease-resistant substances, and leads to decreased plant resistance (Zhang et al., 2022b; Jiang et al., 2023). This study only selected spraying distance and angle as parameters; the selection and refinement of spraying parameters require further research.

Numerous studies have shown that the time after application significantly affects plant growth and physiology (Zhang et al., 2018; Xia et al., 2024). However, Robert et al. (2020) found that the degree of oxidative stress in plants depended more on the dose of the compound added than on the measurement date. Zhang et al. (2018) found no significant differences in some physiological indicators in pepper leaves 7, 14, and 21 days after treatment with different mass concentrations of N-phenylphthalamic acid. This study primarily investigated the effects of pesticide spraying parameters on the change in lesion percentage and physiological responses in leaves of different black spot disease grades, aiming to improve control efficiency, achieve graded precision application, and reduce leaf stress and pesticide pollution. Future research will focus on the influence of time after pesticide application on physiological indicators.

## 5 Conclusion

This study determined the optimal spraying parameters for controlling jujube leaf black spot disease at different severity grades and identified representative physiological indicators highly correlated with control efficiency by analyzing the effects of spraying parameters on control efficiency and leaf physiological indicators. For controlling jujube leaf black spot disease: Grade 0 is best prevented with a spraying distance of 0.9 m and angle of 0°; Grade 1 with 1.2 m and 0°; Grade 2 with 0.6 m and 40°; Grade 3 with 0.6 m and 20°; and Grade 4 with 0.9 m and 40°. Further research found that SS content, SOD and POD activities, and PAL activity were significantly positively correlated with control efficiency. SOD and POD are key physiological indicators for studying the control efficiency of pesticide application on jujube leaf black spot disease across different grades. This study proposes grading-optimized parameters, integrating precision application and physiological impact research, which can reduce pesticide usage and provide technical support for the green control of jujube leaf black spot disease.

## Data Availability

The original contributions presented in the study are included in the article/supplementary material. Further inquiries can be directed to the corresponding authors.
